# Limited effect of reducing pulmonary tuberculosis incidence amid mandatory facial masking for COVID-19

**DOI:** 10.1186/s12931-023-02365-x

**Published:** 2023-02-17

**Authors:** En-Cheng Lin, Hung-Pin Tu, Chien-Hui Hong

**Affiliations:** 1 Department of Dermatology, Kaohsiung Veterans General Hospital, Kaohsiung City, 813414 Taiwan; 2 Department of Dermatology, School of Medicine, National Yang Ming Chiao Tung University, Taipei City, 11221 Taiwan; 3 Department of Public Health and Environmental Medicine, School of Medicine, College of Medicine, Kaohsiung Medical University, Kaohsiung City, 807 Taiwan

**Keywords:** Tuberculosis, Multidrug-resistant tuberculosis, COVID-19

## Abstract

Although the incidence and mortality rates associated with tuberculosis (TB) have been decreasing in many countries, TB remains a major public health concern. Obligatory facial masking and reduced health-care capacity because of COVID-19 may substantially influence TB transmission and care. The Global Tuberculosis Report 2021 published by the World Health Organization indicated a TB rebound at the end of 2020, which coincided with the COVID-19 pandemic. We explored this rebound phenomenon in Taiwan by investigating whether TB incidence and mortality are affected by COVID-19 because of their common route of transmission. In addition, we investigated whether the incidence of TB varies across regions with different incidences of COVID-19. Data (2010–2021) regarding annual new cases of TB and multidrug-resistant TB were collected from the Taiwan Centers for Disease Control. TB incidence and mortality were assessed in Taiwan’s seven administrative regions. Over the last decade, TB incidence decreased continually, even during 2020 and 2021, the years coinciding with the COVID-19 pandemic. Notably, TB incidence remained high in regions with low COVID-19 incidence. However, the overall decreasing trends of TB incidence and mortality remained unchanged during the pandemic. Facial masking and social distancing may prevent COVID-19 transmission but exhibit limited efficacy in reducing TB transmission. Thus, during health-related policymaking, policymakers must consider TB rebound, even in the post-COVID-19 era.

**Supplementary Information:**

The online version contains supplementary material available at 10.1186/s12931-023-02365-x.

## Introduction

Tuberculosis (TB) remains a key public health concern worldwide. The World Health Organization (WHO) reported a global TB incidence of 9.9 million in 2020 [1]. The causative agent of TB is *Mycobacterium tuberculosis* (*MTB*), a highly aerobic bacterium [2]. TB can be active or latent, corresponding to the presence or absence of clinical symptoms, respectively. The typical symptoms of active TB include productive cough with bloody sputum, night sweats, low-grade fever, and weight loss [3, 4]. Although TB can be transmitted through various routes, air transmission is the predominant mode. The process through which TB bacilli are aerosolized is strongly correlated with coughing [5]. TB incidence varies across countries; the highest incidence (≥ 300 cases per 100,000 individuals) is observed in sub-Saharan Africa, whereas low incidences (< 25 cases per 100,000 individuals) are noted in Japan and the United States [6]. Taiwan has an intermediate TB burden; the incidence rates were 72.5 and 45.7 cases per 100,000 individuals in 2005 and 2015, respectively [7, 8].

Because of their specialized cell wall, TB bacilli exhibit increasing levels of drug resistance [9]. To reduce drug resistance, active TB is treated using antibiotic cocktails [10]. However, drug-resistant TB remains a major threat to public health worldwide; the emergence of drug-resistant MTB strains may be attributed to poor drug compliance or low-quality medication use [11]. Multidrug-resistant TB (MDR-TB) is characterized by resistance to the two most effective first-line TB drugs, rifampicin and isoniazid. By contrast, extensively drug-resistant TB (XDR-TB) is characterized by resistance to three or more of the six classes of second-line TB drugs. A study conducted in 2021 defined XDR-TB as TB caused by *MTB* strains resistant to isoniazid, rifampicin, fluoroquinolone, and either bedaquiline or linezolid (or both) [12].

Drug resistance complicates the prevention of TB transmission. Directly observed therapy (DOT) and DOT-Plus (MDR-TB project) have substantially improved drug compliance and medical availability [13]. The WHO reported 480,000 and 201,997 global cases of MDR-TB in 2013 and 2019, respectively. In 2020, the incidence of drug-resistant TB decreased to 157,903. This decrease is consistent with a considerable reduction (18%) in the total number of newly diagnosed TB cases from 2019 to 2020 [14]. MDR-TB has become a concern in Taiwan since 2006, when the rate of drug resistance was higher than that reported by the WHO (third global TB drug resistance surveillance report) [15]. The implementation of the Taiwan Multidrug-resistant Tuberculosis Consortium (TMTC) program with DOTS-Plus effectively reduced the incidence of MDR-TB.

COVID-19, which is caused by SARS-CoV-2, has claimed millions of lives since the end of 2019 [16]. The primary mode of SARS-CoV-2 transmission is exposure to air droplets carrying the pathogen; this is similar to the mode of TB transmission. Droplets of varying sizes are produced during exhalation processes, such as quiet breathing, singing, speaking, coughing, sneezing, and exercise [17–20].

Since the onset of COVID-19, strict protocols have been adopted worldwide to prevent COVID-19 transmission. These measures include facial masking, social distancing, and contact tracing. During the peak of the pandemic, schools and other institutes were closed; gatherings, travel, and movements were restricted; and online activities were promoted [21]. Until May 2021, most cases of COVID-19 in Taiwan were imported cases; however, after this period, an outbreak of local cases in northern Taiwan was reported [22]. Taiwan’s stringent border control policies effectively prevented viral transmission from abroad, and the aforementioned measures curbed the local outbreak.

TB can be transmitted though droplets of varying sizes, from large to fine aerosol particles [23]. Masks such as surgical masks and N95 respirators prevent the entry of contaminated droplets into the respiratory tract. The pandemic facilitated studies regarding the effects of facial masking and social distancing on TB incidence. COVID-19 limited health-care capacity and medical resources. A Korean study exploring the TB notification rate during the COVID-19 pandemic revealed that the rate in 2020 (49/100,000) was the lowest since 2012 in South Korea; the TB notification rate in 2020 was 16.4% lower than that in 2019 [24]. These findings may be attributed to social distancing during the pandemic. In younger individuals, the TB notification rate increased during the pandemic [24]. The aforementioned study inspired us to investigate whether a similar trend occurred in Taiwan during the pandemic. The Global Tuberculosis Report 2021 published by the WHO [1] indicated that TB incidence and mortality had rebounded at the end of 2020. Considering the inconsistency in TB incidence during the COVID-19 pandemic, we investigated the effects of COVID-19 prevention measures (facial masking and social distancing) on TB transmission in Taiwan. In addition, we investigated whether TB mortality, which is associated with care quality, varies across regions with different incidence rates of COVID-19.

## Methods

Demographic data (2010–2021) regarding the annual incidence and mortality rates associated with TB and MDR-TB were collected from a public domain, the Taiwan National Infectious Disease Statistics System (NIDSS), Taiwan Centers for Disease Control (CDC; https://nidss.cdc.gov.tw/; last updated on February 9, 2022) [25–27]. In Taiwan, TB is regarded as a class III notifiable infectious disease. All confirmed cases at each health-care institution must be reported to the Taiwan CDC. Using the NIDSS statistical data, the country was divided into seven regions on the basis of administrative geography: Taipei region; Northern region; Southern region; Central region; Eastern region; Kao-Pin region; and outlying islands, including the Pescadores, Kinmen, and Matsu Islands. The NIDSS data included patients’ residential regions instead of the regions where they were infected. The incidences of TB and MDR-TB were defined as new confirmed cases per 100,000 individuals from the general population.

To estimate the dynamic (national and region-specific) incidence of TB and MDR-TB, general population statistical data (2010–2021; the number of individuals in each region) were obtained from the Taiwan Statistics Bureau [28]. Statistical analysis was performed using Excel (version 2016; Microsoft Corporation, Redmond, WA, USA).

Incidence rate ratios (IRRs) were calculated for the year-wise comparison of incidence rates (reference year: 2010–2011). IRR was calculated through Poisson regression analysis, a log-linear model. Linear trends between IRR and incidence year were calculated using a generalized linear or log-linear model. The data followed a chi-square distribution with 1 degree of freedom. Spearman’s rank correlation analysis was performed to evaluate the strength and direction of the correlation between TB and COVID-19 incidences [29]. All tests were two-sided, and statistical significance was set at *p* < 0.05. All analyses were performed using SAS (version 9.4; SAS Institute, Cary, NC, USA).

## Results

### No additional decrease was noted in TB and MDR-TB incidences during the COVID-19 pandemic

**Fig. 1:**
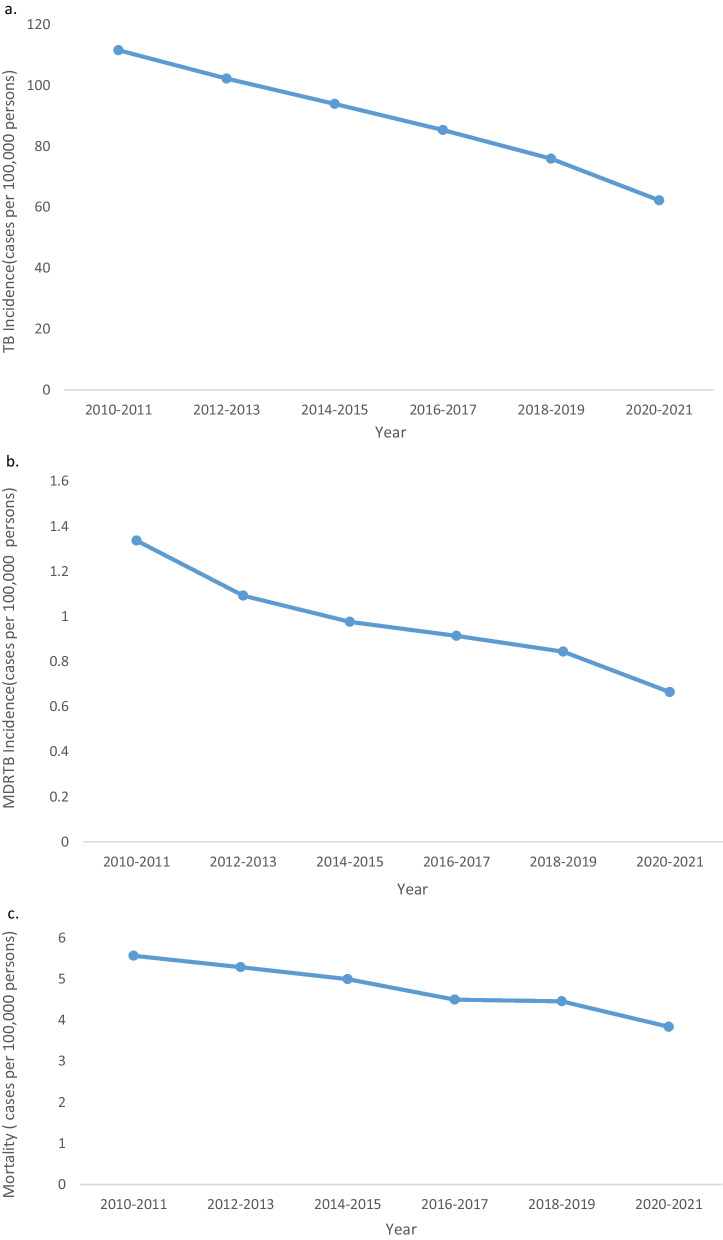
TB and MDR-TB incidence and mortality between 2010 and 2021. **a** TB incidence decreased gradually. **b** MDR-TB incidence decreased continually. **c** TB mortality decreased continually. *TB* tuberculosis; *MDR-TB* multidrug-resistant tuberculosis

Since the beginning of 2020, stringent public health measures have been implemented in Taiwan to prevent COVID-19 transmission. Because most TB types and COVID-19 spread through air and droplets, we investigated whether facial masking and social distancing during the COVID-19 pandemic reduced TB transmission. The decreasing trend in the incidences of TB and MDR-TB in 2020–2021 was similar to that noted in the preceding years (2010–2019; Fig. 1a, b). The incidence of TB continually decreased throughout the study period, and no acceleration was noted in the rate of decrease during the COVID-19 pandemic (Additional file 1: Table S1). The corresponding 95% confidence interval values were overlapped. Likewise, the decreasing trend in the overall TB mortality was not accelerated during the pandemic (Fig. 1c; Additional file 1: Table S3). By the way, the trend of incidence of MDR-TB showed a continuous decline but without statistical significance (Additional file 1: Table S2).

### Incidences of TB and MDR-TB were higher in the Eastern and Southern regions than in other regions

**Fig. 2:**
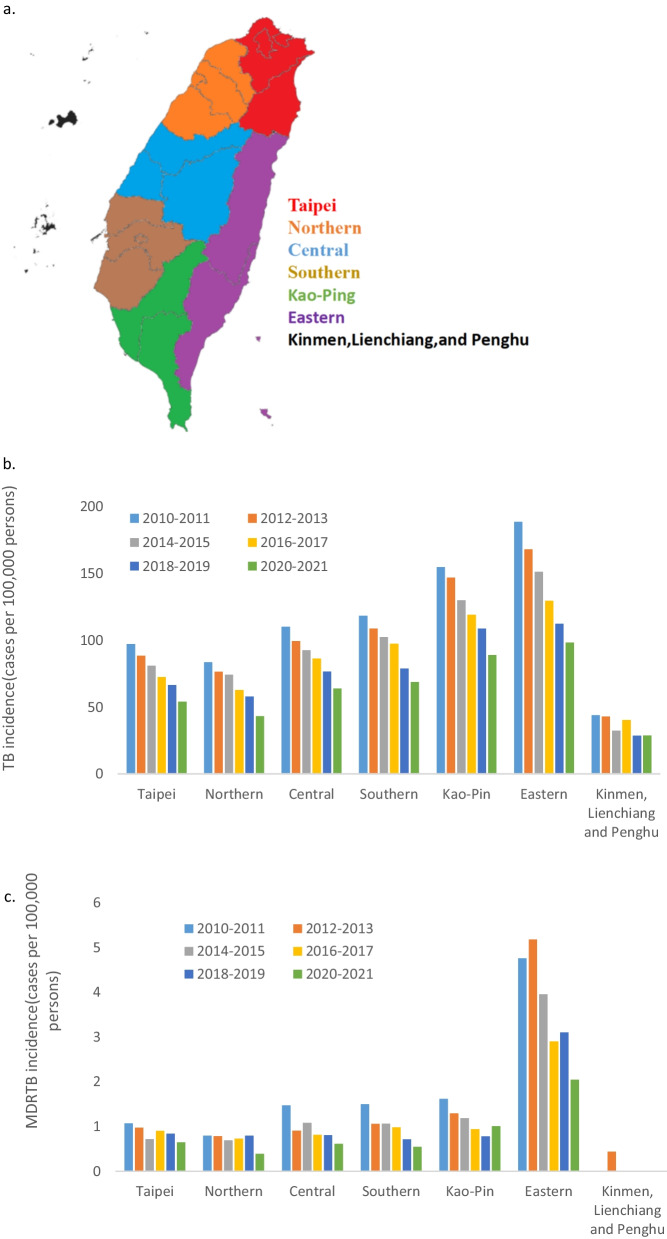
Incidences of TB and MDR-TB across **a** seven regions in Taiwan. **b** TB incidence was the highest in the Eastern region and then in the Kao-Pin region. **c** MDR-TB incidence was also the highest in the Eastern region. *TB* tuberculosis; *MDR-TB* multidrug-resistant tuberculosis

We investigated the correlation between COVID-19 and TB incidences across seven regions in Taiwan. Across the years, TB incidence was the highest in the Eastern region and then in the Kao-Ping region (Fig. 2). Moreover, MDR-TB incidence was the highest in the Eastern region. The IRR values of TB and MDR-TB with overlapping 95% confidence interval values indicated that the incidences of TB and MDR-TB decreased gradually without any acceleration during the COVID-19 pandemic (Additional file 1: Tables S4, S5).

### Geographical distribution of COVID-19 in Taiwan

**Fig. 3:**
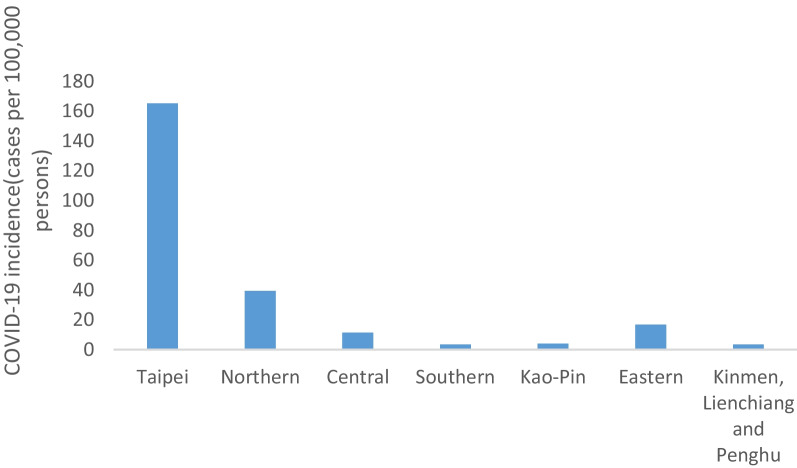
All confirmed cases of COVID-19 across the seven regions. The incidence was the highest in the Taipei region and the lowest in the Kao-Pin region and outlying islands (e.g., Kinmen, Lienchiang, and Penghu)

We speculated the regional incidence of TB might be positively correlated with that of COVID-19. The highest incidence of COVID-19 was observed in the Taipei region, where the capital of Taiwan is located (Fig. 3). By contrast, the lowest incidence COVID-19 was observed in the Southern region, Kao-Pin region, and outlying islands.

### Correlation between TB and COVID-19 incidences across regions

**Fig. 4:**
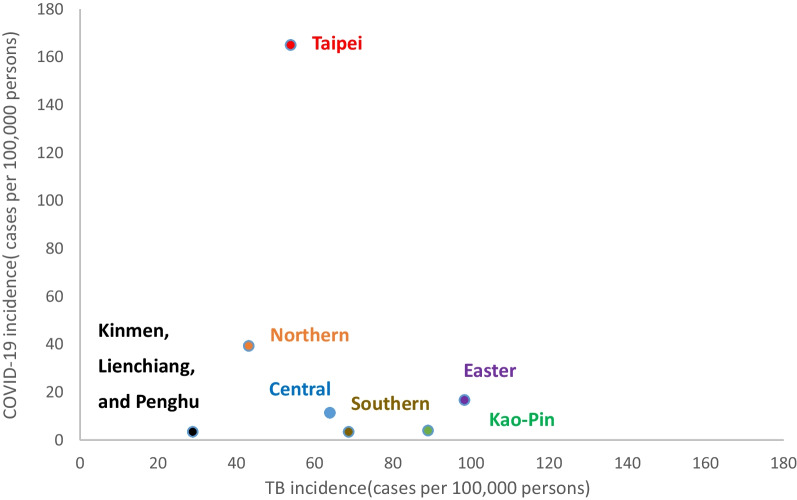
Correlation between TB and COVID-19 incidences stratified by region. Spearman’s rank correlation coefficients were calculated. The regions with low incidences of COVID-19 (e.g., the Kao-Pin region) exhibited high incidences of TB. Thus, the COVID-19 prevention measures may not be effective in preventing TB transmission

Despite the low incidence of COVID-19 in the Kao-Pin and Eastern regions, that of TB remained high. TB incidence did not increase in the Taipei region, where the incidence of COVID-19 was the highest. Although a correlation was noted between TB and COVID-19 incidences, it was not significant [Spearman’s rank correlation coefficient =  − 0.11 (outliers were not considered because of low incidences); Fig. 4; Additional file 1: Table S6]. Although facial masking and social distancing help prevent COVID-19 transmission, they exhibited limited efficacy in preventing TB transmission.

## Discussion

Over the last decade, the incidence of TB continually exhibited a decreasing trend. The decreasing trend in TB incidence and mortality remained unchanged in Taiwan during the COVID-19 pandemic. Thus, the COVID-19 prevention measures may have benefits against TB. Yet, the regions with low incidences of COVID-19 exhibited high incidences of TB.

Considering the high TB burden at the beginning of the twenty-first century in Taiwan, the Taiwan CDC has been promoting the “Mobilization Plan to Halve Tuberculosis Incidence in Ten Years” campaign since 2006 through several strategies, including DOTS [22]. We found that TB incidence continually decreased by approximately 10 cases per 100,000 individuals every 2 years from 2010 to 2021, which includes the pandemic period. This finding is different from that reported by the WHO in the Global Tuberculosis Report 2021 [1], which indicated TB rebound at the end of 2022 despite the limited effects of COVID-19-related disruptions on TB incidence. The sharp decline in TB incidence in 2020 may be attributed to health-care problems and delayed diagnosis in countries with a high TB burden. The changes in the TB notification rates in these countries from 2019 to 2020 might be explained by several factors, such as the onset of the COVID-19 pandemic, severity of the disease, and capacity and resilience of health-care systems. In these countries, the availability of and accessibility to medical care, including TB care, might have been considerably affected by the pandemic. Nevertheless, the health-care system in Taiwan was not substantially affected by the COVID-19 pandemic. Thus, differences were noted between Taiwan and other high-TB-burden countries in terms of TB incidence. In countries with a high TB burden, COVID-19 prevention measures exerted no effects on TB incidence in 2021. However, in one study from Italy, they noticed that the diagnosis of tuberculosis was delayed during first wave of the COVID-19 pandemic [30]. In Korea, Kim et al., found that social distancing practices decreased health care access and tuberculosis notification, particularly among individuals aged 60 years or older. On the contrary, TB notification among younger individuals was increased [24]. Moreover, in another research from Taiwan, they revealed the significant decline of TB activity during fighting against COVID-19 outbreak in Taiwan (during the first 20 weeks of 2020) [31].

The seven-region model of TB incidence in Taiwan revealed a gradual decrease in TB incidence across regions, except for the outlying islands. No sharp decline was noted in the incidence of TB in regions with high (e.g., the Taipei region) or low (e.g., the Kao-Pin region) incidences of COVID-19. Thus, the policies implemented for preventing COVID-19 transmission may not be effective in preventing TB transmission. Another probable reason is the long incubation time of TB (approximately 2–12 weeks), which necessitates long-term studies.

In Taiwan, TB mortality gradually decreased from 2010 to 2021, even during the COVID-19 pandemic. This finding varies from the global data. Studies have reported that the annual TB mortality may increase to that noted in 2015 or 2012 [32, 33]. The WHO (Global Tuberculosis Report 2020) stated that the global increase in TB mortality may result from a decrease in the rate of TB diagnosis (compared with the prepandemic rate) and the number of incidence months [34]. Therefore, TB diagnosis should not be postponed during the pandemic; in addition, anti-TB drugs must be initiated at the earliest time possible. We noted no sharp decline in TB incidence during the pandemic; hence, COVID-19 might not have affected the diagnosis and management of TB in Taiwan.

This study has some limitations. Data regarding patients’ age, comorbidities, socioeconomic status, and other relevant factors were unavailable in the NIDSS and thus were not analyzed in this study. Furthermore, some patients might have had latent TB, which was not diagnosed during the COVID-19 pandemic. Therefore, long-term studies are needed to evaluate TB incidence and mortality.

## Conclusions

During the COVID-19 pandemic, the incidence of TB in countries with a high TB burden sharply declined in 2020 but rebounded immediately in 2021. In Taiwan, TB incidence declined gradually, even during the COVID-19 pandemic. TB mortality increased globally because of delayed diagnosis and treatment; nevertheless, this increase in TB mortality was not observed in Taiwan.

## Supplementary Information

**Additional file 1: Table S1.** Incidence of tuberculosis in Taiwan between 2010 and 2021. **Table S2.** Incidence of multidrug-resistant tuberculosis in Taiwan between 2010 and 2021. **Table S3.** Mortality associated with tuberculosis in Taiwan between 2010 and 2021. **Table S4.** Seven-region model of tuberculosis in Taiwan. **Table S5.** Seven-region model for multidrug-resistant tuberculosis in Taiwan. **Table S6.** Coefficients for the correlation between tuberculosis and COVID-19 incidences.

## Data Availability

Data are available from Taiwan Centers for Diseases Control and Department of Household Registration, Ministry of the Interior, Republic of China (Taiwan).
