# Supplementary material for: Limited effect of reducing pulmonary tuberculosis incidence amid mandatory facial masking for COVID-19
Source: Respir Res. 2023 Feb 17;24:54. doi: 10.1186/s12931-023-02365-x (PMC9936458; doi:10.1186/s12931-023-02365-x)
Supplement: Supplementary file 1 — Additional file 1: Table S1. Incidence of tuberculosis in Taiwan between 2010 and 2021. Table S2. Incidence of multidrug-resistant tuberculosis in Taiwan between 2010 and 2021. Table S3. Mortality associated with tuberculosis in Taiwan between 2010 and 2021. Table S4. Seven-region model of tuberculosis in Taiwan. Table S5. Seven-region model for multidrug-resistant tuberculosis in Taiwan. Table S6. Coefficients for the correlation between tuberculosis and COVID-19 incidences. [file 12931_2023_2365_MOESM1_ESM.docx]

**Additional tables**

**Table S1.** Incidence of tuberculosis in Taiwan between 2010 and 2021

| Year | Total Population | TB new cases | TB incidence rate  (per 100,000 population) | Incidence  rate ratio (95% CI)† | p |
| --- | --- | --- | --- | --- | --- |
| 2010-2011 | 23193517.50 | 25871 | 111.54 | 1.00 (Reference) |  |
| 2012-2013 | 23344669.50 | 23866 | 102.23 | 0.91 (0.70 to 1.19) | 0.4939 |
| 2014-2015 | 23462913.50 | 22037 | 93.92 | 0.83 (0.63 to 1.10) | 0.1901 |
| 2016-2017 | 23531930.50 | 20087 | 85.36 | 0.75 (0.57 to 0.99) | 0.0499 |
| 2018-2019 | 23585319.00 | 17911 | 75.94 | 0.67 (0.50 to 0.90) | 0.0070 |
| 2020-2021 | 23482057.50 | 14619 | 62.26 | 0.55 (0.40 to 0.75) | 0.0002 |
| Incidence rate ratio (95% CI), P-Trend‡ |  |  |  | 0.89 (0.85 to 0.94) | <0.0001 |

†Incidence rate ratio (IRR) was calculated through Poisson regression analysis, a log-linear model.

‡Linear trends between IRR and incidence year were calculated using a generalized linear or log-linear model. The data followed a chi-square distribution with 1 degree of freedom.

**Table S2.** Incidence of multidrug-resistant tuberculosis in Taiwan between 2010 and 2021

| Year | Total Population | MDR-TB new cases | MDR-TB incidence rate  (per 100,000  population) | Incidence  rate ratio (95% CI) | p |
| --- | --- | --- | --- | --- | --- |
| 2010-2011 | 23193517.50 | 310 | 1.34 | 1.00 (Reference) |  |
| 2012-2013 | 23344669.50 | 255 | 1.09 | 0.81 (0.06 to 10.17) | 0.8717 |
| 2014-2015 | 23462913.50 | 229 | 0.98 | 0.72 (0.05 to 9.81) | 0.8066 |
| 2016-2017 | 23531930.50 | 215 | 0.91 | 0.67 (0.05 to 9.64) | 0.7711 |
| 2018-2019 | 23585319.00 | 199 | 0.84 | 0.62 (0.04 to 9.47) | 0.7317 |
| 2020-2021 | 23482057.50 | 156 | 0.66 | 0.49 (0.03 to 9.31) | 0.6355 |
| Incidence rate ratio (95% CI), P-Trend* |  |  |  | 0.88 (0.54 to 1.43) | 0.6065 |

†Incidence rate ratio (IRR) was calculated through Poisson regression analysis, a log-linear model.

‡Linear trends between IRR and incidence year were calculated using a generalized linear or log-linear model. The data followed a chi-square distribution with 1 degree of freedom.

MDR-TB, multidrug resistance tuberculosis.

**Table S3.** Mortality associated with tuberculosis in Taiwan between 2010 and 2021

| Year | Total Population | TB death cases | Mortality rate  per 100,000  population | Mortality  rate ratio (95% CI) | p |
| --- | --- | --- | --- | --- | --- |
| 2010-2011 | 23193517.50 | 1292 | 5.57 | 1.00 (Reference) |  |
| 2012-2013 | 23344669.50 | 1235 | 5.29 | 0.94 (0.29 to 3.10) | 0.9237 |
| 2014-2015 | 23462913.50 | 1173 | 5.00 | 0.89 (0.27 to 2.97) | 0.8459 |
| 2016-2017 | 23531930.50 | 1058 | 4.50 | 0.80 (0.23 to 2.76) | 0.7182 |
| 2018-2019 | 23585319.00 | 1052 | 4.46 | 0.79 (0.23 to 2.74) | 0.7068 |
| 2020-2021 | 23482057.50 | 902 | 3.84 | 0.68 (0.19 to 2.50) | 0.5625 |
| Mortality rate ratio (95% CI), P-Trend* |  |  |  | 0.93 (0.75 to 1.15) | 0.5085 |

†Mortality rate was calculated through Poisson regression analysis, a log-linear model.

‡Linear trends between mortality rate ratio and year were calculated using a generalized linear or log-linear model. The data followed a chi-square distribution with 1 degree of freedom.

**Table S4.** Seven-region model of tuberculosis in Taiwan

| Year | Total Population | TB new cases | TB incidence  rate  per 100,000 population | Incidence  rate ratio (95% CI)† | p |
| --- | --- | --- | --- | --- | --- |
| Taipei |  |  |  |  |  |
| 2010-2011 | 7383583.00 | 7176 | 97.19 | 1.00 (Reference) |  |
| 2012-2013 | 7461547.00 | 6593 | 88.36 | 0.90 (0.67 to 1.20) | 0.4719 |
| 2014-2015 | 7503331.50 | 6068 | 80.87 | 0.82 (0.61 to 1.10) | 0.1842 |
| 2016-2017 | 7502328.00 | 5442 | 72.54 | 0.73 (0.54 to 0.99) | 0.0468 |
| 2018-2019 | 7490840.00 | 4988 | 66.59 | 0.68 (0.49 to 0.92) | 0.0136 |
| 2020-2021 | 7408881.50 | 3993 | 53.89 | 0.55 (0.40 to 0.77) | 0.0005 |
| Incidence rate ratio (95% CI), P-Trend‡ |  |  |  | 0.90 (0.85 to 0.95) | <0.0001 |
| Northern |  |  |  |  |  |
| 2010-2011 | 3502197.50 | 2923 | 83.46 | 1.00 (Reference) |  |
| 2012-2013 | 3555873.50 | 2718 | 76.44 | 0.90 (0.66 to 1.23) | 0.5148 |
| 2014-2015 | 3620436.00 | 2683 | 74.11 | 0.86 (0.63 to 1.17) | 0.3407 |
| 2016-2017 | 3691831.00 | 2315 | 62.71 | 0.71 (0.51 to 0.99) | 0.0427 |
| 2018-2019 | 3755259.00 | 2174 | 57.89 | 0.65 (0.46 to 0.90) | 0.0109 |
| 2020-2021 | 3830083.50 | 1653 | 43.16 | 0.47 (0.33 to 0.68) | <0.0001 |
| Incidence rate ratio (95% CI), P-Trend‡ |  |  |  | 0.87 (0.82 to 0.93) | <0.0001 |
| Central |  |  |  |  |  |
| 2010-2011 | 4486218.00 | 4941 | 110.14 | 1.00 (Reference) |  |
| 2012-2013 | 4509926.50 | 4484 | 99.43 | 0.90 (0.68 to 1.18) | 0.4367 |
| 2014-2015 | 4534315.50 | 4197 | 92.56 | 0.83 (0.63 to 1.10) | 0.1907 |
| 2016-2017 | 4558077.50 | 3936 | 86.35 | 0.77 (0.58 to 1.02) | 0.0714 |
| 2018-2019 | 4574124.00 | 3505 | 76.63 | 0.68 (0.51 to 0.91) | 0.0102 |
| 2020-2021 | 4566409.50 | 2920 | 63.95 | 0.57 (0.42 to 0.78) | 0.0004 |
| Incidence rate ratio (95% CI), P-Trend‡ |  |  |  | 0.90 (0.86 to 0.95) | <0.0001 |
| Southern |  |  |  |  |  |
| 2010-2011 | 3403534.50 | 4029 | 118.38 | 1.00 (Reference) |  |
| 2012-2013 | 3394340.00 | 3691 | 108.74 | 0.92 (0.71 to 1.19) | 0.5360 |
| 2014-2015 | 3380342.50 | 3463 | 102.45 | 0.87 (0.67 to 1.14) | 0.3075 |
| 2016-2017 | 3365580.50 | 3275 | 97.31 | 0.83 (0.64 to 1.09) | 0.1769 |
| 2018-2019 | 3350963.00 | 2641 | 78.81 | 0.68 (0.51 to 0.90) | 0.0071 |
| 2020-2021 | 3307340.50 | 2273 | 68.73 | 0.60 (0.44 to 0.80) | 0.0007 |
| Incidence rate ratio (95% CI), P-Trend‡ |  |  |  | 0.91 (0.86 to 0.95) | <0.0001 |
| Kao-Pin |  |  |  |  |  |
| 2010-2011 | 3642995.50 | 5641 | 154.85 | 1.00 (Reference) |  |
| 2012-2013 | 3634631.50 | 5341 | 146.95 | 0.95 (0.76 to 1.19) | 0.6639 |
| 2014-2015 | 3623540.00 | 4706 | 129.87 | 0.84 (0.67 to 1.06) | 0.1518 |
| 2016-2017 | 3613550.00 | 4304 | 119.11 | 0.78 (0.61 to 0.98) | 0.0369 |
| 2018-2019 | 3602080.00 | 3915 | 108.69 | 0.71 (0.56 to 0.91) | 0.0062 |
| 2020-2021 | 3567695.00 | 3176 | 89.02 | 0.59 (0.45 to 0.76) | <0.0001 |
| Incidence rate ratio (95% CI), P-Trend‡ |  |  |  | 0.90 (0.87 to 0.94) | <0.0001 |
| Eastern |  |  |  |  |  |
| 2010-2011 | 567303.00 | 1070 | 188.61 | 1.00 (Reference) |  |
| 2012-2013 | 560080.00 | 941 | 168.01 | 0.90 (0.73 to 1.11) | 0.3323 |
| 2014-2015 | 556129.50 | 841 | 151.22 | 0.82 (0.66 to 1.01) | 0.0655 |
| 2016-2017 | 551445.50 | 714 | 129.48 | 0.71 (0.56 to 0.88) | 0.0023 |
| 2018-2019 | 548014.00 | 615 | 112.22 | 0.62 (0.49 to 0.78) | <0.0001 |
| 2020-2021 | 537907.00 | 529 | 98.34 | 0.55 (0.43 to 0.70) | <0.0001 |
| Incidence rate ratio (95% CI), P-Trend‡ |  |  |  | 0.89 (0.85 to 0.92) | <0.0001 |
| Kinmen, Lienchiang and Penghu |  |  |  |  |  |
| 2010-2011 | 207686.00 | 91 | 43.82 | 1.00 (Reference) |  |
| 2012-2013 | 228271.00 | 98 | 42.93 | 0.89 (0.59 to 1.36) | 0.5926 |
| 2014-2015 | 244818.50 | 79 | 32.27 | 0.62 (0.40 to 0.98) | 0.0426 |
| 2016-2017 | 250615.00 | 101 | 40.30 | 0.76 (0.50 to 1.17) | 0.2135 |
| 2018-2019 | 255364.00 | 73 | 28.59 | 0.53 (0.33 to 0.85) | 0.0084 |
| 2020-2021 | 260025.50 | 75 | 28.84 | 0.53 (0.33 to 0.84) | 0.0073 |
| Incidence rate ratio (95% CI), P-Trend‡ |  |  |  | 0.88 (0.81 to 0.95) | 0.0012 |

†Incidence rate ratio (IRR) was calculated through Poisson regression analysis, a log-linear model.

‡Linear trends between IRR and incidence year were calculated using a generalized linear or log-linear model. The data followed a chi-square distribution with 1 degree of freedom.

**Table S5.** Seven-region model for multidrug-resistant tuberculosis in Taiwan

| Year | Total Population | TB new cases | TB incidence  rate  per 100,000 population | Incidence  rate ratio (95% CI)† | p |
| --- | --- | --- | --- | --- | --- |
| Taipei |  |  |  |  |  |
| 2010-2011 | 7383583.00 | 79 | 1.07 | 1.00 (Reference) |  |
| 2012-2013 | 7461547.00 | 73 | 0.98 | 0.90 (0.06 to 14.04) | 0.9430 |
| 2014-2015 | 7503331.50 | 54 | 0.72 | 0.66 (0.03 to 13.14) | 0.7866 |
| 2016-2017 | 7502328.00 | 68 | 0.91 | 0.83 (0.05 to 13.68) | 0.8986 |
| 2018-2019 | 7490840.00 | 63 | 0.84 | 0.77 (0.04 to 13.48) | 0.8610 |
| 2020-2021 | 7408881.50 | 48 | 0.65 | 0.60 (0.03 to 13.20) | 0.7483 |
| Incidence rate ratio (95% CI), P-Trend‡ |  |  |  | 0.92 (0.56 to 1.54) | 0.7609 |
| Northern |  |  |  |  |  |
| 2010-2011 | 3502197.50 | 28 | 0.80 | 1.00 (Reference) |  |
| 2012-2013 | 3555873.50 | 28 | 0.79 | 0.97 (0.04 to 21.79) | 0.9847 |
| 2014-2015 | 3620436.00 | 25 | 0.69 | 0.84 (0.03 to 20.91) | 0.9129 |
| 2016-2017 | 3691831.00 | 27 | 0.73 | 0.87 (0.04 to 20.69) | 0.9302 |
| 2018-2019 | 3755259.00 | 30 | 0.80 | 0.93 (0.04 to 20.70) | 0.9644 |
| 2020-2021 | 3830083.50 | 15 | 0.39 | 0.45 (0.01 to 20.48) | 0.6805 |
| Incidence rate ratio (95% CI), P-Trend‡ |  |  |  | 0.91 (0.52 to 1.59) | 0.7319 |
| Central |  |  |  |  |  |
| 2010-2011 | 4486218.00 | 66 | 1.47 | 1.00 (Reference) |  |
| 2012-2013 | 4509926.50 | 41 | 0.91 | 0.61 (0.04 to 8.4) | 0.7153 |
| 2014-2015 | 4534315.50 | 49 | 1.08 | 0.73 (0.06 to 8.71) | 0.8011 |
| 2016-2017 | 4558077.50 | 37 | 0.81 | 0.54 (0.04 to 8.16) | 0.6588 |
| 2018-2019 | 4574124.00 | 37 | 0.81 | 0.54 (0.04 to 8.13) | 0.6555 |
| 2020-2021 | 4566409.50 | 28 | 0.61 | 0.41 (0.02 to 8.06) | 0.5569 |
| Incidence rate ratio (95% CI), P-Trend‡ |  |  |  | 0.86 (0.53 to 1.40) | 0.5430 |
| Southern |  |  |  |  |  |
| 2010-2011 | 3403534.50 | 51 | 1.50 | 1.00 (Reference) |  |
| 2012-2013 | 3394340.00 | 36 | 1.06 | 0.71 (0.06 to 8.54) | 0.7870 |
| 2014-2015 | 3380342.50 | 36 | 1.06 | 0.72 (0.06 to 8.58) | 0.7918 |
| 2016-2017 | 3365580.50 | 33 | 0.98 | 0.66 (0.05 to 8.44) | 0.7506 |
| 2018-2019 | 3350963.00 | 24 | 0.72 | 0.49 (0.03 to 8.11) | 0.6149 |
| 2020-2021 | 3307340.50 | 18 | 0.54 | 0.37 (0.02 to 8.31) | 0.5341 |
| Incidence rate ratio (95% CI), P-Trend‡ |  |  |  | 0.84 (0.52 to 1.37) | 0.4936 |
| Kao-Pin |  |  |  |  |  |
| 2010-2011 | 3642995.50 | 59 | 1.62 | 1.00 (Reference) |  |
| 2012-2013 | 3634631.50 | 47 | 1.29 | 0.80 (0.08 to 8.07) | 0.8502 |
| 2014-2015 | 3623540.00 | 43 | 1.19 | 0.74 (0.07 to 7.87) | 0.8003 |
| 2016-2017 | 3613550.00 | 34 | 0.94 | 0.59 (0.05 to 7.43) | 0.6798 |
| 2018-2019 | 3602080.00 | 28 | 0.78 | 0.49 (0.03 to 7.25) | 0.6004 |
| 2020-2021 | 3567695.00 | 36 | 1.01 | 0.64 (0.05 to 7.64) | 0.7214 |
| Incidence rate ratio (95% CI), P-Trend‡ |  |  |  | 0.89 (0.57 to 1.39) | 0.6003 |
| Eastern |  |  |  |  |  |
| 2010-2011 | 567303.00 | 27 | 4.76 | 1.00 (Reference) |  |
| 2012-2013 | 560080.00 | 29 | 5.18 | 1.10 (0.32 to 3.83) | 0.8785 |
| 2014-2015 | 556129.50 | 22 | 3.96 | 0.85 (0.22 to 3.22) | 0.8084 |
| 2016-2017 | 551445.50 | 16 | 2.90 | 0.63 (0.15 to 2.70) | 0.5311 |
| 2018-2019 | 548014.00 | 17 | 3.10 | 0.67 (0.16 to 2.82) | 0.5898 |
| 2020-2021 | 537907.00 | 11 | 2.04 | 0.45 (0.09 to 2.33) | 0.3438 |
| Incidence rate ratio (95% CI), P-Trend‡ |  |  |  | 0.86 (0.67 to 1.10) | 0.2234 |
| Kinmen, Lienchiang and Penghu |  |  |  |  |  |
| 2010-2011 | 207686.00 | 0 | 0.00 | 1.00 (Reference) |  |
| 2012-2013 | 228271.00 | 1 | 0.44 | - | - |
| 2014-2015 | 244818.50 | 0 | 0.00 | - | - |
| 2016-2017 | 250615.00 | 0 | 0.00 | - | - |
| 2018-2019 | 255364.00 | 0 | 0.00 | - | - |
| 2020-2021 | 260025.50 | 0 | 0.00 | - | - |
| Incidence rate ratio (95% CI), P-Trend‡ |  |  |  | - | - |

-, not calculated.

†Incidence rate ratio (IRR) was calculated through Poisson regression analysis, a log-linear model.

‡Linear trends between IRR and incidence year were calculated using a generalized linear or log-linear model. The data followed a chi-square distribution with 1 degree of freedom.

MDR-TB, multidrug-resistant tuberculosis.

**Table S6.** Coefficients for the correlation between tuberculosis and COVID-19 incidences

|  | TB incidence rate | P-value |
| --- | --- | --- |
| Pearson correlation (r) |  |  |
| COVID-19 incidence rate | -0.21 | 0.6455 |
| Spearman's rank correlation (ρ) |  |  |
| COVID-19 incidence rate | -0.11 | 0.8192 |

Pearson correlation analysis was performed to evaluate the strength and direction of the correlation between tuberculosis (TB) and COVID-19 incidences in 2020 and 2021.

Spearman’s rank correlation analysis was performed to evaluate the strength and direction of the correlation between TB and COVID-19 incidences in 2020 and 2021.
